# Middlesex Hospital

**Published:** 1831-12

**Authors:** 


### HOSPITAL REPORTS.

### MIDDLESEX HOSPITAL.

*Clinical Lecture on Hernia, delivered November 5th, by* Herbert Mayo, f.r.s.

Gentlemen: A portion of intestine contained in a hernial sac is said to be strangulated, when the neck of the sac is so narrow as to obstruct, more or less completely, the passage of the contents of the bowel. It rarely happens that in strangulated hernia the circulation of the blood in the strangulated part is in any great degree obstructed. The first symptoms of a strangulated hernia are, a tumor which presents under different circumstances very different characters; more or less uneasiness and tenderness in the tumor, and in the adjacent region of the belly; vomiting, and constipation.

In the treatment of strangulated hernia, the most dangerous fault is delay in operating, when other means have proved ineffectual. The fatal results of such delay are frequently illustrated in hospitals, into which patients with hernia are continually brought, two, three, or four days after strangulation has begun: in many of these cases the operation, though performed without loss of time after the admission of the patient, does not save his life. In the patient on whom I operated in the hospital last week, the femoral hernia, under which he laboured, had been strangulated from Friday to the Tuesday following: the symptoms at this time did not appear to be grave: the tumor was indeed painful on pressure, but it was not particularly tense, and it was difficult to detect decided tenderness of the belly; the patient had not vomited lately, nor was there hiccup; nor was the countenance anxious, nor the tongue furred, nor the pulse materially affected. But the taxis had already been tried by the house-surgeon, and I feared the consequences of delay, and immediately performed the operation.

In addition to a portion of thickened omentum, the sac was found to contain a coil of small intestine, about five inches long: this was not inflamed, nor in a state approaching to gangrene, but it was dark with venous congestion: it was returned into the abdomen. The patient experienced considerable relief. He now took some broth, which he retained on his stomach, and in the afternoon began taking some gentle aperient medicine, by which the bowels in a few hours were freely moved: nevertheless, an obscure tenderness of the belly on pressure, which had never wholly left the patient, and for which leeches were applied on the evening after the operation and repeated subsequently with other appropriate remedies, after a few hours greatly increased; the tongue became dry, the pulse frequent and wiry, the belly tympanitic, and, in sixty hours from the operation, the patient died. On opening the body after death, the peritoneum was found highly inflamed; the folds of intestine near the part which had been strangulated were glued together with recent lymph, and the part itself was of the same dark colour it had shewn when in the hernial sac; it had not mortified, but had been sufficiently injured by the long period of strangulation to cause fatal peritoneal inflammation.

Had the patient lived a few days longer, partial mortification of the strangulated part might have taken place. Four or five years ago, an old man, in Hertford ward, on whom I operated for hernia, and in whom the bowel appeared during the operation in the condition which I had described, died ten days afterwards with inflammation of the peritoneum. In this instance the dark-coloured intestine had mortified in two patches after its return into the belly.

I may take this opportunity of noticing the following remarkable feature in surgical pathology. Inflammations of serous membranes, of the peritoneum and pleura at least, when produced by mechanical causes, are commonly insidious in their progress, and are not attended with that local distress and general febrile derangement which attend the like complaints when idiopathic. In illustration of this remark, I may mention the case of a boy, fourteen years of age, whose body you saw examined at the same time with that of the hernia patient whose case I have above described: he had lived forty-eight hours after being kicked on the lower part of the belly by a horse. The only symptom observed by those who attended him till within a few hours of his death, when we learn that some tenderness of the belly supervened, was occasional vomiting. Nevertheless, the ileum had been ruptured a few inches above the valvuli coli; alimentary matter had passed into the cavity of the belly; and the most intense inflammation, with effusion of purulent serum and large adherent flakes of lymph, was found, upon opening the body, to have occurred. The same insidious march of peritoneal inflammation may frequently be detected in hernia.

It is principally in hospital practice, or amongst the lower orders, that you witness cases of hernia in which strangulation has existed many hours, and in which the dark-coloured bowel, which presents itself on opening the sac, has been *so long* in a state of congestion that, when it is returned into the belly, it but serves to excite or heighten peritoneal inflammation.

In a case of this description, (an inguinal hernia, in a patient forty-two years of age,) occurring in the Middlesex Hospital, I opened the discoloured bowel, and, returning all but the part adjacent to the opening, attached the latter by a ligature to the integument: the patient had not a bad symptom; the contents of the bowels passed for a time by the artificial anus, but subsequently were brought to pass along their proper channel, and in about three months from the operation the aperture in the groin was entirely healed. This patient is Mr. John Quick, of 72, Titchfield street, a very respectable tradesman: after his recovery, he made a donation of ten guineas to the hospital, and became an annual subscriber.*

In a second case, in which I tried the same method, the patient died; but he already laboured under decided peritonitis when brought into the hospital.

In the preceding observations, as far as they relate to the treatment of hernia, I have illustrated the ill consequences of delay in operating, when the fault has not been attributable to the operator: let me now suppose or describe cases in which the operation for strangulated hernia has been delayed, or its necessity overlooked by the surgeon.

A patient may have two ruptures at the same abdominal opening: one may be smaller than and behind the other, and be strangulated, while the other may admit of easy reduction. An incautious practitioner, called to such a case, may be satisfied with reducing the more obvious hernia, and leave the patient to die of the other, attributing the subsequent continuance of the symptoms to some internal cause of strangulation. A gentleman, who was house-surgeon to this hospital, communicated to me the following case, which occurred in his time: A patient had strangulated inguinal hernia; he was bled, and placed in the warm bath, and, after much and long-continued kneeding, the tumor disappeared; but the symptoms continued, and the patient died. Upon examining the body, it was found that the hernia had indeed been pressed out of the spermatic passage, but not out of the sac; in other words, both the hernia and the stricture were returned, and the taxis, in this instance, instead of relieving the complaint, had only fatally obscured its nature.

The result of the following case I witnessed myself: A surgeon had been called to a young woman, labouring under strangulated crural hernia; the tumor had yielded to the pressure which he had

* We know not why Mr. Mayo has omitted to mention that this hospital patient made *him* a present of a silver tankard, which, with a laudable pride, he displays at his hospitable board.—Editor.

made, and he was satisfied that a little *flat* lump which remained was the sac alone, covered by the inguinal fat, and glands, and fascia; but the symptoms continued unrelieved, and the patient died. I was present at the opening of the body: a small flat portion of the bowel, (a portion only of its cylinder,) had remained nipped and strangulated in the sac, which had caused the death of the patient.

One rule of practice may be clearly deduced from the preceding cases. When you have had to deal with a hernial tumor attended with symptoms of strangulation, and the tumor has yielded entirely or in great part to the taxis, but the symptoms still continue, do not hesitate to cut down to the sac, in order to ascertain whether any cause of strangulation yet remains, which you can relieve.

One of the commonest symptoms of strangulated hernia is constipation of the bowels; and this symptom is seemingly so essential a result of strangulation, that a surgeon may be led by its absence alone to fatal delay: but it is evident that the hernial sac may contain a diverticulum of the intestine, or it may nip a *portion only* of the cylinder of the bowel, or the sac may contain omentum alone. In either of these cases it may be necessary to operate, although the passage of the bowels be not completely obstructed.

The same anomaly, the absence of constipation in strangulated hernia, may result from another cause: the stricture at the neck of the sac may be narrow enough to produce the other symptoms of hernia and death, without being so close as wholly to prevent a passage. The following unfortunate case is an instance of this: Towards the end of September, a patient, about forty years of age, was admitted into the hospital under my care: he was suffering with considerable disorder of the stomach and bowels; he vomited occasionally, and occasionally suffered from acute colic pains; the bowels acted. On the right side there was a collection of fluid in the scrotum, which had every appearance of being a hydrocele: it extended to the external ring, and the upper part, which was as usual narrower than the rest, seemed to have more consistence than the bag of fluid below. The account which this patient gave of his case was contradictory, but he led us to suppose that the hydrocele, as it seemed to be, of the right testis had been punctured by a surgeon some time before, and had filled again; and he said, and unsaid afterwards, that the upper and firmer part of the swelling occurred upon his making some bodily exertion two days before his admission. There was no tenderness of the belly above the tumor; the tumor itself was scarcely tender on pressure at the upper part: I thought, however, when the patient coughed, that I could occasionally distinguish a faint pulsation communicated to the tumor from the abdomen. However, the united opinion of my colleagues that the case was hydrocele with enlargement of the chord, led me to give up (at least not to act upon) my own impression, that the upper part of the tumor was a hernia. Some warm opening medicines were therefore administered, and leeches and fomentations were ordered to be applied to the abdomen: these measures gave some relief. An injection was likewise given, and the bowels acted. It was the second day after this, that the symptoms, although in some degree abated, continuing, and the patient complaining of pain in the scrotum, I opened the lower part of the tumor with a lancet: a serous fluid, of the usual character in hydrocele, was evacuated, and left an enlarged mass in the lower part of the scrotum, which resembled a diseased testis, from which to the ring there extended what might be thought a thickened spermatic cord. This operation gave the patient some relief; the previous symptoms, however, still continued, and stercoraceous vomiting supervened: nevertheless, the bowels acted as before, upon the administration of a dose of castor oil. This circumstance continued to corroborate the impression that the case was not a case of strangulated hernia. Notwithstanding the ambiguity which the absence of this symptom occasioned, my colleagues agreed with me that, under the circumstances I have described, it would be right to lay open the upper part of the tumor. I did this, and found in a hernial sac a long narrow fold of small intestine, scarcely darker than natural, and with so little stricture at the neck of the sac, that I returned the bowel without dividing the stricture. The operation, however, was performed too late; the patient sank, and died.

Upon the examination of the parts, the hernia was found to have been of the following complicated description: there had been a congenital omental hernia; the middle of the length of protruded omentum had at a subsequent period become adherent to the cord, so as to isolate the chamber of the serous cavity in which the testis lay from the chamber communicating with the belly. A hydrocele had formed in the isolated chamber. That which had been supposed to be an enlarged testicle, was the end of the omentum in contact with the testis, and greatly enlarged and lobulated. The omentum above this part was thickened up to the ring, so as to present to the touch the character of a thickened spermatic cord.

